# Nighttime versus Fulltime Brace Treatment for Adolescent Idiopathic Scoliosis: Which Brace to Choose? A Retrospective Study on 358 Patients

**DOI:** 10.3390/jcm12247684

**Published:** 2023-12-14

**Authors:** Vojtech Capek, Adad Baranto, Helena Brisby, Olof Westin

**Affiliations:** 1 Department of Orthopedics, Sahlgrenska University Hospital, SE413 45 Gothenburg, Swedenolof.westin@vgregion.se (O.W.); 2 Institute of Clinical Sciences, Sahlgrenska Academy, University of Gothenburg, SE413 45 Gothenburg, Sweden

**Keywords:** brace treatment, nighttime brace treatment, adolescent idiopathic scoliosis, scoliosis, Providence brace, Boston brace

## Abstract

The purpose of this study is to retrospectively compare the effectiveness of fulltime Boston Brace (BB) and Providence Nighttime Brace (PNB) treatments in moderate scoliotic curves (20–40°) at a single institution and to carry out analyses for different subgroups. Inclusion criteria: idiopathic scoliosis, age ≥ 10 years, curve 20–40°, Risser ≤ 3 or Sanders stage ≤ 6 and curve apex below T6 vertebra. Exclusion criteria: incomplete radiological or clinical follow-up and previous treatment. The primary outcome was failure according to the SRS outcome assessment: increase in main curve > 5° and/or increase in main curve beyond 45° and/or surgery. The subgroup analyses were secondary outcomes. In total, 249 patients in the PNB and 109 in the BB groups were included. The BB showed a higher success rate compared to the PNB (59% and 46%, respectively) in both crude and adjusted comparisons (*p* = 0.029 and *p* = 0.007, respectively). The subgroup analyses showed higher success rates in pre-menarchal females, thoracic curves and curves > 30° in the BB group compared to the PNB group. Based on the findings, fulltime braces should be the treatment of choice for more immature patients and patients with larger and thoracic curves while nighttime braces might be sufficient for post-menarchal females and patients with lumbar and smaller curves.

## 1. Introduction

**Figure 1:**
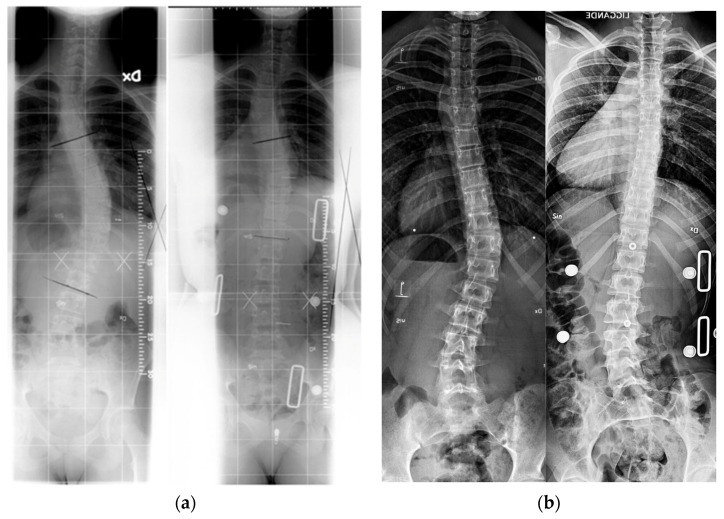
Pre-brace and in-brace radiographs for Boston and Providence brace treatments: (**a**) left: pre-brace (40° curve); right: in Boston brace (17°); (**b**) left: pre-brace (31° curve); right: overcorrection in Providence brace (−4°).

Adolescent idiopathic scoliosis (AIS) mostly affects healthy adolescent females [1]. The yearly incidence is 3–5%; however, only 0.3–0.5% require treatment. In AIS, the growing spine starts to rotate shortly before the growth spurt and creates a typical lateral scoliotic curve. The deterioration usually continues until full maturity is reached and can lead to a severe spine deformity if it is left untreated. Meanwhile, severe scoliotic deformities (radiologic Cobb angle > 45°) are treated surgically, and milder forms of progressive deformities (20–40°) can respond to brace treatment [2,3]. Although there are many different brace types and designs, their common goal is to align the spine during the growth spurt by external forces and limit the progression of the scoliotic curve (Figure 1).

A traditional fulltime brace (e.g., Boston brace) is sought to be worn for 18–23 h per day, even though some treatment effect is still noticeable after 12 h of daily wear [2,4]. The treatment is demanding for the patient as well as their family, and adherence to the treatment varies substantially (33–93%) [5,6]. Nighttime braces (e.g., Providence, Charleston) are supposed to limit the negative psycho-social effects of brace treatment and increase adherence to the treatment while maintaining equal treatment effect. Even though early studies showed promising results on nighttime bracing, later works showed inconclusive results, with treatment success ranging from 15% to 93% [7,8,9,10,11,12,13,14,15,16,17]. This discrepancy might be due to different inclusion criteria, outcome measures for success, compliance assessment and/or maturity staging. Therefore, new guidelines for brace studies in AIS patients were created by the Scoliosis Research Society (SRS) Committee [18]. However, only a few studies on nighttime bracing have attempted to follow these guidelines [9,10,13,14].

Here, we present what is to our knowledge the largest study comparing treatment effectiveness in 358 consecutive AIS patients treated either with a fulltime Boston brace (BB) or a Providence nighttime brace (PNB) at a single institution. Both the SRS assessment criteria of effectiveness for brace studies and the increase in the Cobb angle until final follow-up were primary outcome measures for treatment effectiveness. In addition, compliance as well as treatment effectiveness in different subgroups were compared as secondary outcome measures.

## 2. Materials and Methods

### 2.1. Study Design

**Figure 2:**
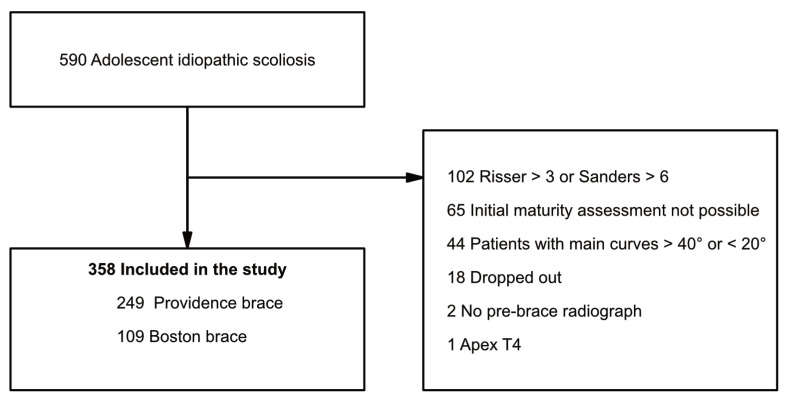
Study flowchart.

A retrospective observational case–control study approved by the Swedish Ethical Review Authority (DN: 2020-04364) was conducted at a single institution specialized in the treatment of spinal deformity. The patients were identified within the records of the institution’s Orthotics Department, where all orthotic/brace treatments performed at the clinic were registered. Patients treated between 2003 and 2019 were included. Although the BB was gradually replaced by the PNB in the 2010s, the indication criteria for brace treatment have remained the same over time. The inclusion criteria were idiopathic scoliotic curves 20–40° Cobb angle, age over 10 years, Risser stage ≤ 3 and/or Sanders stage ≤ 6 and apex of the main curve below T6 vertebra. Exclusion criteria were incomplete clinical and radiological follow-up or previous treatment for scoliosis. In total, 590 consecutive AIS patients from the Orthotics Department’s records were cross-referenced with the medical records and reviewed. Of these, 102 patients were excluded due to advanced radiological skeletal maturity, 85 patients due to missing radiological or clinical data, 44 patients due to having curves >40° or <20° and 1 patient due to having apex at the T4 vertebra. Thus, 358 patients were included in the study (Figure 2).

### 2.2. Treatment Procedure

Both studied braces, the Boston brace (BB; Allard UK Ltd., Dunalk, Ireland) and the Providence nighttime brace (PNB; Spinal Technology, LLC, West Yarmouth, MA, USA), are semi-custom-made devices; their treatment principles, measurement and manufacture are described elsewhere [7,19]. The BB is a fulltime brace intended for 23 h of daily wear, whereas the PNB is prescribed for night-wear only. After the brace was fitted, the radiographs were taken to ensure an acceptable (50%) in-brace correction (i.e., supine or standing radiographs in PNB or BB patients, respectively). Brace adjustments were usually necessary during the first 4–6 weeks to reach an optimal fit. Clinical and/or radiological follow-ups were taken every 6–12 months depending on the risk of curve progression. The brace was checked for a good fit at each visit by a dedicated team consisting of a spine surgeon, physiotherapist and orthotic engineer. Any treatment changes and compliance issues were documented. The treatment was discontinued when skeletal maturity was reached (Risser ≥ 4 and/or Sanders ≥ 7 and/or 2 years after menarche). At least one post-treatment follow-up visit was performed about 1 year after discontinuation of the treatment.

### 2.3. Outcome Measures and Variables

The primary outcome measure was determined based on the recommendation of the SRS Committee on Bracing and Nonoperative Management [18]. Thus, treatment failure was defined as: (1) increase in the scoliotic curve by ≥6° before the 1-year follow-up visit and/or (2) increase in the curve beyond 45° and/or (3) surgery being recommended or performed within 2 years after discontinuation of the treatment. The brace groups were also compared with respect to increase in the main curve during the treatment period. A simplified Lenke classification was used for five major curve patterns: Major Thoracic, Double Thoracic, Double Major, Triple Major and Thoracolumbar/Lumbar (Lenke 5 and 6). In the case of double curves, the larger curve was labeled as the primary curve.

Since inclusion in the study stretched over a prolonged period of time, the Risser skeletal maturity assessment was gradually replaced by the Sanders staging system. In order to enable comparison between different maturity assessments, a modified Sanders stage (MSS) variable was introduced. This variable was constructed in agreement with the previous work by Sanders et al. [20] and consists of either a true Sanders stage, where available, or a Risser stage changed to a Sanders stage, i.e., MSS 3 ≈ Risser 0 and pelvic triradiate cartilage (TC) open, MSS 4 ≈ Risser 0 and TC closing, MSS 5 ≈ Risser 0 and TC closed, MSS 6 ≈ Risser 1–3, MSS 7 ≈ Risser 4, MSS 8 ≈ Risser 5. The radiographs of 76 patients included in a previously published randomized study were assessed independently by two observers [21]. The remaining radiographs were assessed by one individual. Additionally, a per-protocol analysis was performed excluding patients whose initial brace treatment was changed to a different one. Finally, an analysis of certain subgroups was performed, and the compliance was compared between the groups.

### 2.4. Compliance

A physiotherapist specialized in brace treatment took records of patients’ well-being, compliance and brace-fit at each follow-up visit. Based on these records, the patients were retrospectively divided into four groups: excellent, good, fair and poor. The patients with excellent compliance throughout the whole treatment or those with minor compliance issues during less than 25% of the prescribed time were assigned to the “excellent” group. The patients with minor compliance issues in less than half of the prescribed time were assigned to the “good” group, e.g., a PNB patient who involuntarily “opened up” the brace on fewer than half of the nights or a BB patient who was not able to wear the brace at school. The patients who had major compliance issues were assigned to the “fair” group, e.g., a PNB patient who was not able to wear the brace more than 3 nights/week, a BB patient who was able to use the brace during nights only or any patient who discontinued the treatment after less than half of the predicted treatment period. All patients who discontinued bracing after less than 25% of the prescribed time or used the brace less than 25% of the daily recommendation were assigned to the “poor” group.

### 2.5. Statistical Analyses

The main results were presented as proportions of treatment failures/successes in each brace group and/or a mean difference in outcome between the groups. The 95% confidence intervals (95% CI) were bootstrapped or robust where normal distribution or homoscedasticity were violated, respectively. Both crude and adjusted differences in outcome between the brace groups were calculated. A multivariable logistic regression analysis was performed for dichotomous outcome measures adjusted for propensity score. Similarly, analysis of covariance (ANCOVA) was used for comparison of adjusted continuous data. Significantly different variables at baseline with effect size > 0.2 were selected as covariates/factors for adjusted calculations. The within-groups point estimates of continuous variables were presented as means (SD) and medians (IQR—interquartile range). Student’s t-test was used for continuous and Fischer’s exact test for dichotomous variables between groups. The Mann–Whitney U test and Chi square test were used for ordered and non-ordered categorical data, respectively. The significance tests were two-sided at the 5% significance level. The statistical analysis was performed using SPSS v 28.0 (SPSS Inc., Chicago, IL, USA).

## 3. Results

### 3.1. Study Population

Age at bracing, gender, delay to bracing, proportion of pre-menarchal females and curve characteristics were similar in both groups (Table 1). Patients in the BB group had significantly larger initial main curves, longer follow-up and inferior in-brace correction (IBC). A majority of patients presented with thoracic curves followed by the group with TL/L curves. The MSS was significantly different between the groups (*p* = 0.042), i.e., the MSS 6 was overrepresented in the PNB group. Of 358 patients included in the study, 88% were females, and more than half of these were pre-menarche. The patients were treated for a mean of 1.9 years and followed up for a mean of 1.5 years after treatment cessation.

### 3.2. Main Outcome

The overall success rates according to the SRS criteria were 46% and 59% in PNB and BB groups, respectively, and they differed significantly in favor of the Boston brace (*p* = 0.029; Table 2). In order to take into account other variables that might influence the outcome, a logistic regression was performed with failure according to the SRS assessment criteria as the dependent variable adjusted for the propensity score. The initial curve magnitude and MSS were selected as factors in the propensity score based on the baseline differences for these variables. The other baseline characteristics, e.g., menarche, age and type of the curve, lacked both the statistical significance and effect size and, therefore, were not included in the model. In the adjusted analysis, the risk for failure after being treated by the PNB was 1.91 times higher (OR: 1.91 [1.19, 3.05]) than if treated by the BB (*p* = 0.007; Table 2). Surgery was either performed or recommended within 2 years after brace treatment cessation in 28.9% and 22.9% of patients in the PNB and BB groups, respectively (*p* = 0.30). When adjusted for propensity score, the patients in the PNB group had 1.93 times significantly higher odds for surgery than patients in the BB group (95% CI [1.08, 3.42]; *p* = 0.025; Table 2).

The main curve in the PNB group increased more during the treatment until the 1-year follow-up compared to in the BB group (Table 3). The average increase in Cobb angle during the treatment period was 7.3° and 4.5° in the PNB and BB groups, respectively (*p* = 0.009). Subsequently, the Analysis of Covariance (ANCOVA) model was built with the same covariates as in the former analysis, i.e., the initial Cobb angle and MSS 1–3 vs. MSS 4–7. Comparing the groups, the difference in the curve progression after adjustment was 3.1° with 95% CI [1.1°, 5.1°], showing a significantly worse outcome in the PNB group (*p* = 0.002; Table 3).

### 3.3. Per Protocol Analysis

In total, 12 (3.4%) patients crossed over to another brace group, i.e., 9 BB patients moved to the PNB group, 1 PNB patient moved to the BB group and 2 patients started treatment with another type of brace. Thus, the per protocol analysis was performed on the remaining 346 patients. The ANCOVA for adjusted means comparison and the multivariable logistic regression were performed with the same covariates as in the main analysis. The crossovers did not have any impact on the main results. The adjusted mean difference of the main curve progression until follow-up was 3.0° [0.9°, 5.1°], showing less progression in the BB group. The multivariable logistic regression with adjustment for propensity score showed 1.8 times higher odds for failure in the PNB group compared to the BB group (95% CI [1.1, 2.9]; *p* = 0.013), thus showing similar results as in the main analysis.

### 3.4. Secondary Outcomes

The subgroup analysis was performed for certain subgroups selected by gender, menarchal status, type of main curve and curve magnitude (Table 4). Both outcomes, i.e., failure according to the SRS and increase in the main curve until follow-up, were calculated. The BB performed better in females compared to males, in pre-menarchal females, in patients with thoracic curves and in those with 30–40° curves. On the other hand, the two braces performed similarly in males, post-menarchal females and patients with lumbar curves. In the patient subgroup with minor curves (20–29°), the PNB showed a larger increase in the curve angle, although there was no significant difference in odds for failure between the groups. The patients in the PNB group, further, showed significantly better compliance with the treatment (Table 5), whereas 74% of the PNB patients had excellent compliance while only 55% in the BB group did.

## 4. Discussion

### 4.1. Main Results

This, to our knowledge, is the largest study comparing fulltime and nighttime bracing for AIS at a single institution. Further, it is the first brace study attempting to compare two different treatments using the two most common outcome measures, i.e., the failure rates based on the SRS recommendation and the increase in the main curve at follow-up. Interestingly, both outcome measures converged toward similar results. The BB showed overall superior performance compared to the PNB. Within different subgroups, the BB showed a better outcome in pre-menarchal females and patients with larger curves and thoracic curves. Moreover, in an attempt to control for potential confounders, the results were adjusted for important differences in baseline characteristics between the groups.

Previously published studies comparing nighttime and fulltime bracing showed different success rates ranging between 15% and 78% in the fulltime group and 31% and 73% in the nighttime group [9,10,13,15,21]. In contrast to the present study, however, no significant differences in treatment success between the groups were found. The possible explanation might be a limited ability to detect a difference in treatments, lack of data on skeletal maturity and/or failure to account for other predictors influencing the outcome in these studies. In the present study, the mature patients were rigorously excluded, and the covariates were methodically selected based on baseline differences between the groups.

The treatment success rates of 45% and 59% for the PNB and BB, respectively, are poorer than expected. The rigorous inclusion criteria, high proportion of pre-menarchal females in both groups and application of SRS outcome assessment criteria might explain these results. Indeed, in the study by Janicki et al., where the SRS outcome criteria were first applied, the results were even more disappointing, with treatment success rates of 15% and 31% in fulltime (TLSO) and nighttime (PNB) groups, respectively [13]. On the other hand, those studies using a certain cutoff value for failure, e.g., the curve exceeding 45° magnitude or the need for surgery, usually present higher success rates for brace treatment [2,15].

### 4.2. Secondary Outcomes

The main result of the comparisons within the subgroups was that the fulltime brace might better protect against failure in more immature patients, thoracic curves and larger curves compared to the nighttime brace, whereas patients with lumbar curves and more mature patients, e.g., post-menarchal females, might be treated by either of the braces. This finding is supported by the meta-analysis by Buyuk et al., who carefully concluded that nighttime bracing might be an alternative to fulltime bracing in Risser 1 or 2 patients with lumbar curves [22]. Indeed, a pioneer study by D’Amato showed excellent results in PNB treatment especially for lumbar curve patterns (94% success) [7]. Also, the BB performed better in the treatment of patients with curves > 30° regarding both outcomes, while the results for curves < 30° were not conclusive. This finding is in agreement with those of previous studies showing that patients with larger initial curves are at a higher risk of failing brace treatment [23,24]. Therefore, more intensive (i.e., fulltime) brace treatment might be warranted.

Compliance with the treatment is a crucial prerequisite for treatment success [2,25,26]. In the present study, the PNB patients demonstrated significantly better compliance with the treatment than the BB patients. This finding is supported by other studies where nighttime braces were generally better accepted than fulltime braces, even though the range of good compliance between the studies is wide (49–93% nighttime, 33–73% fulltime) [5,27,28,29,30,31,32,33]. Surprisingly, the BB in the current study performed better despite poorer compliance. This might be due to an absolute amount of time spent in the brace. The results of our study suggest that nighttime bracing might be an insufficient treatment in certain highly progressive curves even with good compliance.

### 4.3. Limitations

This is a retrospective study, and, thus, 67 (16%) patients were excluded due to an incomplete radiological assessment. Moreover, 18 (4%) patients dropped out from the follow-up. Also, despite the mean follow-up in the PNB and BB groups of 1.4 and 1.6 years, respectively, not all of the patients reached a minimum of 1-year follow-up. Nevertheless, the average growth velocity after brace discontinuation was 0.5 cm per year, and the mean age at follow-up was 16.4 years and 17.4 year for females and males, respectively. Also, one of the three criteria for failed treatment was that the surgery had to be performed or recommended within two years after the treatment discontinuation. Since an average follow-up was less than two years, some patients could have been lost to follow-up and/or operated at another hospital. However, all the patients that showed progress of scoliosis in mature age or those whose scoliotic curves had progressed close to the cut-off for surgery (45° Cobb angle) were further followed. Moreover, our institution is the only scoliosis center in the region. Due to the rules for the reimbursement system between the regions, it is unlikely that a significant number of patients would undergo the surgery outside the region.

Next, we could not adhere to the SRS recommendations on objective compliance measurement due to the prolonged time period spanned by this study. Instead, we attempted to categorize the patients according to the subjective reports obtained at each visit. Thus, the compliance is not an exact objective measure, although we believe that the brace groups were comparable, which was the purpose of the categorization. Lastly, due to the change of skeletal maturity assessments along the course of the data collection, we attempted to match the Risser sign to the Sanders staging system, which might have added some bias.

### 4.4. Clinical Implications

The findings of the current study provide additional guidance for decision-making in AIS brace treatment. At our institution, the PNB has been the brace of choice for all AIS patients since the 2010s, irrespective the skeletal maturity stage, curve type, curve magnitude or menarche. However, based on the current results, we must reconsider our treatment standards. We now think that the fulltime brace should be the treatment of choice for more immature patients and larger curves, especially in combination with stiffer thoracic curves. On the other hand, other factors, such as compliance and patients’ psychological and emotional status and preferences, should be taken into account when counselling the family. Although it is not possible to generalize our results, we encourage caution regarding nighttime bracing in general as the PNB’s performance was inferior even with good compliance and excellent IBC. It seems that highly progressive curves close to the peak height velocity need to be addressed more aggressively with fulltime regimes irrespective of chronological age. The PNB can probably still be offered to more mature patients and to non-compliant patients as an alternative for treatment of lumbar and smaller curves.

## 5. Conclusions

The Boston fulltime brace is more effective than the Providence nighttime brace in the treatment of adolescent idiopathic scoliosis in pre-menarchal female patients, patients with thoracic curves and patients with curves > 30° Cobb angle.

## Figures and Tables

**Table 1:** Baseline characteristics for patients in Providence and Boston brace treatments groups.

	Total (*n* = 358)	Providence (*n* = 249)	Boston (*n* = 109)	*p*	Effect Size
Age at bracing	13.41 (1.28) 13.42 (12.53; 14.35)	13.42 (1.32) 13.43 (12.50;14.38)	13.41 (1.21) 13.38 (12.57; 14.28)	0.49	0.003
Gender					
Female	314 (87.7%)	215 (86.3%)	99 (90.8%)		
Male	44 (12.3%)	34 (13.7%)	10 (9.2%)	0.30	0.06
Initial Cobb angle main curve	31.1 (4.7) 31 (27; 35)	30.7 (4.5) 30 (27; 34)	32.1 (4.9) 32.5 (28; 36)	**0.006**	−0.29
Delay to bracing (years)	0.20 (0.08) 0.19 (0.15; 0.24)	0.20 (0.06) 0.19 (0.15; 0.24)	0.19 (0.11) 0.17 (0.13; 0.23)	0.56	0.08
Time spent in brace (years) *	1.91(0.94) 1.81 (1.28; 2.42) *n* = 295	1.87 (0.90) 1.69 (1.28; 2.31) *n* = 195	2.00 (1.01) 1.98 (1.34; 2.53) *n* = 100	0.12	−0.15
Follow up after finishing bracing (years) *	1.45 (0.73) 1.19 (1.02; 1.64) *n* = 295	1.39 (0.66) 1.15 (1.00; 1.53) *n* = 195	1.56 (0.86) 1.25 (1.04; 1.94) *n* = 100	**0.045**	−0.23
Initial modified Sanders stage					
2	7 (2.0%)	3 (1.2%)	4 (3.7%)		
3	48 (13.4%)	31 (12.4%)	17 (15.6%)		
4	109 (30.4%)	74 (29.7%)	35 (32.1%)		
5	58 (16.2%)	37 (14.9%)	21 (19.3%)		
6	135 (37.7%)	104 (41.8%)	31 (28.4%)		
7	1 (0.3%)	0 (0.0%)	1 (0.9%)	**0.042**	
Risser 0	164 (45.8%)	109 (43.8%)	55 (50.5%)		
Risser > 0	194 (54.2%)	140 (56.2%)	54 (49.5%)	0.25	0.062
Main curve size					
Cobb 20–29°	142 (39.7%)	106 (42.6%)	36 (33.0%)		
Cobb 30–40°	216 (60.3%)	143 (57.4%)	73 (67%)	0.10	0.089
Type of curve					
MT (Lenke 1)	169 (47.5%)	125 (50.2%)	44 (40.4%)		
DT (Lenke 2)	29 (8.1%)	18 (7.5%)	11 (10.1%)		
DM (Lenke 3)	37 (10.3%)	22 (8.8%)	15 (13.8%)		
TM (Lenke 4)	1 (0.3%)	1 (0.4%)	0 (0.0%)		
TL/L (Lenke 5 and 6)	122 (34.1%)	83 (33.3%)	39 (35.8%)	0.32	0.11
Menarche at bracing					
Pre-menarche	173 (55.6%)	119 (55.9%)	54 (55.1%)		
Post-menarche	138 (44.4%) *n* = 311	94 (44.1%) *n* = 213	44 (44.9%) *n* = 98	0.90	0.007
In-brace main curve correction (%)	54.7 (23.8) 55.8 (38.5; 71.4)	63.3 (20.2) 64.0 (50.0; 76.7)	34.9 (19.2) 33.3 (19.2; 46.9)	**<0.0001**	−1.4

For categorical variables, *n* (%) is presented. For continuous variables, Mean (SD)/Median (IQR) is presented. Mann–Whitney U test was used for ordered categorical variables, X^2^ test for non-ordered categorical variables, Fischer’s exact test for dichotomous variables and t-test for continuous variables. MT—major thoracic, DT—double thoracic, DM—double major, TM—triple major, TL/L—thoracolumbar/lumbar. Effect sizes are presented as Cohen’s d and Cohen’s w for continuous and categorical variables, respectively. * Only those who did not interrupt the treatment or underwent early surgery are included.

**Table 2:** Primary analysis: comparison of treatment failure/success according to the SRS criteria for the Providence group and the Boston group.

	Providence	Boston			Adjusted Analysis		
			Mean D (95% CI)	*p* Value	OR (95% CI)	*p* Value *	Effect Size **
Total, *n*	249	109					
Failure, *n*	135 (54.2%)	45 (41.3%)	12.9% (1.8, 24.0)	**0.029**	1.91 (1.19, 3.05)	**0.007**	0.12
Success, *n*	114 (45.8%)	64 (58.7%)	−12.9% (−24.0, −1.8)				
Surgery							
Yes	72 (28.9%)	25 (22.9%)	6% (−3.7,15.7)	0.30	1.93 (1.08, 3.42)	**0.025**	0.06
No	177 (71.1%)	84 (77.1%)	−6% (−15.7,3.7)				

SRS criteria for failure were as follows: increase in the main curve by > 5° at follow-up or main curve reaching 45° or surgery. Fischer’s exact test was used for crude comparisons. OR [95% CI] is the odds ratio for failure in the Providence treatment (1) compared to the Boston treatment (0) adjusted for propensity score. Propensity score is the predicted probability of being treated by the Providence brace adjusted for the initial curve magnitude and Modified Sanders stage 1–3. D = difference * Adjusted *p* value. ** Effect size of crude comparison is Cohen’s W.

**Table 3:** Comparison of crude and adjusted means of main curve progression from the start of treatment to the 1-year follow-up.

	Providence		Boston					
	Crude Mean (SD) Median (IQR)	Adjusted Means * (95% CI)	Crude Mean (SD) Median (IQR)	Adjusted Means * (95% CI)	*p*	Adjusted *p* *	Difference between Groups Adjusted Means (95% CI)	Effect Size **
Cobb angle change (°) of main curve	7.3 (9.3) 6.0 (0.5; 12.5) *n* = 249	7.4 (6.2, 8.5) *n* = 249	4.5 (9.1) 3.0 (−2; 10) *n* = 109	4.3 (2.6, 6.0) *n* = 109	**0.009**	**0.002**	3.1 (1.1, 5.1)	0.30

* For adjusted means, Analysis of Covariance (ANCOVA) was used with Initial Cobb angle of main curve and Modified Sanders stage 1–3 as baseline covariates. The confidence interval for adjusted mean difference is robust because heteroscedasticity was assumed. ** Effect size is Cohen’s d of crude means. For crude comparison between groups, *t*-test was used. *p* value is two-sided, significance level 0.05.

**Table 4:** Subgroup analyses—comparison of the main results from the start of treatment to 1-year follow-up in selected subgroups.

		Failure SRS Rates				Adjusted Increase in Cobb Angle		
Selected Subgroup	Brace Group	Proportion of Failures	Mean Difference between Groups [95% CI]	OR [95% CI] *	p *	Within Groups Mean (°) [95% CI] **	Difference between Groups Adjusted Means [95% CI] **	p **
Females	Providence *n* = 215	117 (54.4%)	15.0% [3.3, 24.7]	2.0 [1.2, 3.3]	**0.005**	7.1 (5.9, 8.3)	3.2 (1.1, 5.2)	**0.002**
	Boston *n* = 99	39 (39.4%)				3.9 (2.2, 5.7)		
Males	Providence *n* = 34	18 (52.9%)	−7.1% [−41.8, 27.6]	1.1 [0.2, 5.3]	0.88	9.0 (5.2, 12.7)	1.3 (−8.3, 10.9)	0.79
	Boston *n* = 10	6 (60.0%)				7.7 (0.3, 15.0)		
Pre-menarche	Providence *n* = 119	78 (65.5%)	17.4% [1.6, 33.2]	2.2 [1.1, 4.3]	**0.02**	9.8 (8.1, 11.5)	4.8 (1.8, 7.8)	**0.002**
	Boston *n* = 54	26 (48.1%)				5.0 (2.4, 7.6)		
Post-menarche	Providence *n* = 94	38 (40.4%)	13.1% [−3.4, 29.6]	2.0 [0.9, 4.4]	0.095	3.7 (2.3, 5.1)	1.3 (−1.5, 4.1)	0.34
	Boston *n* = 44	12 (27.3%)				2.4 (0.3, 4.5)		
Thoracic curve	Providence *n* = 155	96 (61.9%)	18.0% [3.8, 32.2]	2.4 [1.3, 4.3]	**0.005**	8.8 (7.3, 10.4)	3.6 (0.9, 6.2)	**0.008**
	Boston *n* = 66	29 (43.9%)				5.3 (3.0, 7.6)		
Lumbar curve	Providence *n* = 94	39 (41.5%)	4.3% [−13.2, 21.8]	1.3 [0.6, 2.8]	0.47	4.9 (3.3, 6.5)	2.2 (−0.8, 5.1)	0.15
	Boston *n* = 43	16 (37.2%)				2.8 (0.4, 5.2)		
Curves 20–29°	Providence *n* = 106	55 (51.9%)	13.0% (−5.6, 31.6)	2.3 (1.0, 5.4)	0.05	8.1 (6.3, 9.9)	3.8 (0.3, 7.3)	**0.034**
	Boston *n* = 36	14 (38.9%)				4.3 (1.1, 7.4)		
Curves 30–40°	Providence *n* = 143	80 (55.9%)	13.4% (−0.6, 27.4)	1.9 (1.04, 3.3)	**0.036**	6.9 (5.4, 8.3)	2.7 (0.2, 5.2)	**0.035**
	Boston *n* = 73	31 (42.5%)				4.2 (2.2, 6.2)		

* OR = odds ratio for SRS failure of Providence brace treatment (1) compared to Boston (0) brace treatment in multivariable logistic regression with propensity score adjustment. ** Analysis of Covariance (ANCOVA) was used with adjustment for Modified Sanders stage 1–3 and Initial Cobb angle. The confidence intervals for ANCOVA were robust where homoscedasticity was not assumed. *p* value is two-sided, significance level 0.05.

**Table 5:** Comparison of compliance between the groups.

	Providence (*n* = 243)	Boston (*n* = 109)	*p*
Compliance			
Excellent	180 (74.1%)	60 (55.0%)	
Good	32 (13.2%)	15 (13.8%)	
Fair	15 (6.2%)	18 (16.5%)	
Poor	16 (6.6%)	16 (14.7%)	**<0.001**

For categorical variables, *n* (%) is presented. For comparison between groups, Mann–Whitney U test was used for ordered categorical variables.

## Data Availability

The original dataset is available within the Supplementary Materials.

## Supplementary Materials

The following supporting information can be downloaded at: https://www.mdpi.com/article/10.3390/jcm12247684/s1, File S1. Original dataset.

## References

[1] Angevine P.D., Deutsch H. (2008). Idiopathic scoliosis. Neurosurgery.

[2] Weinstein S.L., Dolan L.A., Wright J.G., Dobbs M.B. (2013). Effects of bracing in adolescents with idiopathic scoliosis. N. Engl. J. Med..

[3] Nachemson A.L., Peterson L.E. (1995). Effectiveness of treatment with a brace in girls who have adolescent idiopathic scoliosis. A prospective, controlled study based on data from the Brace Study of the Scoliosis Research Society. J. Bone Joint Surg. Am..

[4] Katz D.E., Durrani A.A. (2001). Factors that influence outcome in bracing large curves in patients with adolescent idiopathic scoliosis. Spine.

[5] Antoine L., Nathan D., Laure M., Briac C., Jean-Francois M., Corinne B. (2020). Compliance with night-time overcorrection bracing in adolescent idiopathic scoliosis: Result from a cohort follow-up. Med. Eng. Phys..

[6] MacLean W.E., Green N.E., Pierre C.B., Ray D.C. (1989). Stress and coping with scoliosis: Psychological effects on adolescents and their families. J. Pediatr. Orthop..

[7] D’Amato C.R., Griggs S., McCoy B. (2001). Nighttime bracing with the Providence brace in adolescent girls with idiopathic scoliosis. Spine.

[8] Price C.T., Scott D.S., Reed F.R., Sproul J.T., Riddick M.F. (1997). Nighttime bracing for adolescent idiopathic scoliosis with the Charleston Bending Brace: Long-term follow-up. J. Pediatr. Orthop..

[9] Bohl D.D., Telles C.J., Golinvaux N.S., Basques B.A., DeLuca P.A., Grauer J.N. (2014). Effectiveness of Providence nighttime bracing in patients with adolescent idiopathic scoliosis. Orthopedics.

[10] Davis L., Murphy J.S., Shaw K.A., Cash K., Devito D.P., Schmitz M.L. (2019). Nighttime bracing with the Providence thoracolumbosacral orthosis for treatment of adolescent idiopathic scoliosis: A retrospective consecutive clinical series. Prosthet. Orthot. Int..

[11] Gepstein R., Leitner Y., Zohar E., Angel I., Shabat S., Pekarsky I., Friesem T., Folman Y., Katz A., Fredman B. (2002). Effectiveness of the Charleston bending brace in the treatment of single-curve idiopathic scoliosis. J. Pediatr. Orthop..

[12] Howard A., Wright J.G., Hedden D. (1998). A comparative study of TLSO, Charleston, and Milwaukee braces for idiopathic scoliosis. Spine.

[13] Janicki J.A., Poe-Kochert C., Armstrong D.G., Thompson G.H. (2007). A comparison of the thoracolumbosacral orthoses and providence orthosis in the treatment of adolescent idiopathic scoliosis: Results using the new SRS inclusion and assessment criteria for bracing studies. J. Pediatr. Orthop..

[14] Lee C.S., Hwang C.J., Kim D.J., Kim J.H., Kim Y.T., Lee M.Y., Yoon S.J., Lee D.H. (2012). Effectiveness of the Charleston night-time bending brace in the treatment of adolescent idiopathic scoliosis. J. Pediatr. Orthop..

[15] Ohrt-Nissen S., Lastikka M., Andersen T.B., Helenius I., Gehrchen M. (2019). Conservative treatment of main thoracic adolescent idiopathic scoliosis: Full-time or nighttime bracing?. J. Orthop. Surg..

[16] Simony A., Beuschau I., Quisth L., Jespersen S.M., Carreon L.Y., Andersen M.O. (2019). Providence nighttime bracing is effective in treatment for adolescent idiopathic scoliosis even in curves larger than 35 degrees. Eur. Spine J..

[17] Wiemann J.M., Shah S.A., Price C.T. (2014). Nighttime bracing versus observation for early adolescent idiopathic scoliosis. J. Pediatr. Orthop..

[18] Richards B.S., Bernstein R.M., D’Amato C.R., Thompson G.H. (2005). Standardization of criteria for adolescent idiopathic scoliosis brace studies: SRS Committee on Bracing and Nonoperative Management. Spine.

[19] Emans J.B., Kaelin A., Bancel P., Hall J.E., Miller M.E. (1986). The Boston bracing system for idiopathic scoliosis. Follow-up results in 295 patients. Spine.

[20] Sanders J.O., Khoury J.G., Kishan S., Browne R.H., Mooney J.F., Arnold K.D., McConnell S.J., Bauman J.A., Finegold D.N. (2008). Predicting scoliosis progression from skeletal maturity: A simplified classification during adolescence. J. Bone Joint Surg. Am..

[21] Capek V., Westin O., Brisby H., Wessberg P. (2022). Providence nighttime brace is as effective as fulltime Boston brace for female patients with adolescent idiopathic scoliosis: A retrospective analysis of a randomized cohort. N. Am. Spine Soc. J..

[22] Buyuk A.F., Truong W.H., Morgan S.J., Snyder A.J., Miller D.J., Nolin K.K., Smith K.J. (2021). Is nighttime bracing effective in the treatment of adolescent idiopathic scoliosis? A meta-analysis and systematic review based on scoliosis research society guidelines. Spine Deform..

[23] Karol L.A., Virostek D., Felton K., Jo C., Butler L. (2016). The Effect of the Risser Stage on Bracing Outcome in Adolescent Idiopathic Scoliosis. J. Bone Joint Surg. Am..

[24] Charles Y.P., Canavese F., Dimeglio A. (2017). Curve progression risk in a mixed series of braced and nonbraced patients with idiopathic scoliosis related to skeletal maturity assessment on the olecranon. J. Pediatr. Orthop. B.

[25] Aulisa A.G., Giordano M., Falciglia F., Marzetti E., Poscia A., Guzzanti V. (2014). Correlation between compliance and brace treatment in juvenile and adolescent idiopathic scoliosis: SOSORT 2014 award winner. Scoliosis.

[26] Sanders J.O., Newton P.O., Browne R.H., Katz D.E., Birch J.G., Herring J.A. (2014). Bracing for idiopathic scoliosis: How many patients require treatment to prevent one surgery?. J. Bone Joint Surg. Am..

[27] Morton A., Riddle R., Buchanan R., Katz D., Birch J. (2008). Accuracy in the prediction and estimation of adherence to bracewear before and during treatment of adolescent idiopathic scoliosis. J. Pediatr. Orthop..

[28] Takemitsu M., Bowen J.R., Rahman T., Glutting J.J., Scott C.B. (2004). Compliance monitoring of brace treatment for patients with idiopathic scoliosis. Spine.

[29] Rahman T., Borkhuu B., Littleton A.G., Sample W., Moran E., Campbell S., Rogers K., Bowen J.R. (2010). Electronic monitoring of scoliosis brace wear compliance. J. Child. Orthop..

[30] Donzelli S., Zaina F., Negrini S. (2012). In defense of adolescents: They really do use braces for the hours prescribed, if good help is provided. Results from a prospective everyday clinic cohort using thermobrace. Scoliosis.

[31] Miller D.J., Franzone J.M., Matsumoto H., Gomez J.A., Avendano J., Hyman J.E., Roye D.P., Vitale M.G. (2012). Electronic monitoring improves brace-wearing compliance in patients with adolescent idiopathic scoliosis: A randomized clinical trial. Spine.

[32] Hasler C.C., Wietlisbach S., Buchler P. (2010). Objective compliance of adolescent girls with idiopathic scoliosis in a dynamic SpineCor brace. J. Child. Orthop..

[33] Vandal S., Rivard C.H., Bradet R. (1999). Measuring the compliance behavior of adolescents wearing orthopedic braces. Issues Compr. Pediatr. Nurs..

